# Alterations in the gut microbiota and metabolite profiles of patients with Kashin-Beck disease, an endemic osteoarthritis in China

**DOI:** 10.1038/s41419-021-04322-2

**Published:** 2021-10-28

**Authors:** Xi Wang, Yujie Ning, Cheng Li, Yi Gong, Ruitian Huang, Minhan Hu, Blandine Poulet, Ke Xu, Guanghui Zhao, Rong Zhou, Mikko J. Lammi, Xiong Guo

**Affiliations:** 1 Department of Occupational and Environmental Health, School of Public Health, Xi’an Jiaotong University Health Science Center, Xi’an, Shaanxi 710061 P. R. China; 2 School of Public Health, Xi’an Jiaotong University Health Science Center, Key Laboratory of Trace Elements and Endemic Diseases, National Health and Family Planning Commission, Xi’an, Shaanxi 710061 P. R. China; 3 Global Health Institute, Xi’an Jiaotong University Health Science Center, Xi’an, Shaanxi 710061 P. R. China; 4 Shaanxi Institute of Endemic Disease Prevention and Control, Xi’an, Shaanxi 710003 P. R. China; 5 Institute of Lifecourse and Medical Sciences, Department of Musculoskeletal and Ageing Science, University of Liverpool, 6 West Derby Street, Liverpool, L7 8TX UK; 6 Department of Joint Surgery, Hong Hui Hospital, Xi’an Jiaotong University, No. 555, Youyi East Road, Xi’an, China; 7 Department of Integrative Medical Biology, University of Umeå, Umeå, Sweden

**Keywords:** Metagenomics, Metabolomics

## Abstract

Kashin-Beck disease (KBD) is a severe osteochondral disorder that may be driven by the interaction between genetic and environmental factors. We aimed to improve our understanding of the gut microbiota structure in KBD patients of different grades and the relationship between the gut microbiota and serum metabolites. Fecal and serum samples collected from KBD patients and normal controls (NCs) were used to characterize the gut microbiota using 16S rDNA gene and metabolomic sequencing via liquid chromatography-mass spectrometry (LC/MS). To identify whether gut microbial changes at the species level are associated with the genes or functions of the gut bacteria in the KBD patients, metagenomic sequencing of fecal samples from grade I KBD, grade II KBD and NC subjects was performed. The KBD group was characterized by elevated levels of *Fusobacteria* and *Bacteroidetes*. A total of 56 genera were identified to be significantly differentially abundant between the two groups. The genera *Alloprevotella*, *Robinsoniella*, *Megamonas*, and *Escherichia_Shigella* were more abundant in the KBD group. Consistent with the 16S rDNA analysis at the genus level, most of the differentially abundant species in KBD subjects belonged to the genus *Prevotella* according to metagenomic sequencing. Serum metabolomic analysis identified some differentially abundant metabolites among the grade I and II KBD and NC groups that were involved in lipid metabolism metabolic networks, such as that for unsaturated fatty acids and glycerophospholipids. Furthermore, we found that these differences in metabolite levels were associated with altered abundances of specific species. Our study provides a comprehensive landscape of the gut microbiota and metabolites in KBD patients and provides substantial evidence of a novel interplay between the gut microbiome and metabolome in KBD pathogenesis.

## Introduction

Kashin-Beck disease (KBD) is an endemic degenerative osteochondrosis with irreversible pathological and clinical development, including shortened and enlarged fingers, deformed limb joints, and limited movement [1, 2]. Although the processes involving metabolism, apoptosis, adaptive immune defense, the cytoskeleton, cell movement, and extracellular matrix turnover [3–7] have been found to play key roles during chondrocyte injury in KBD, there are currently no clear underlying mechanisms involved in the occurrence and development of KBD, thus effective treatment options are very limited.

Accumulating evidence suggest that cartilage damage in patients with KBD is driven by the interaction between genetic and environmental factors [8]. Recently, it has been suggested that changes in the gut microbiome composition and metabolic activity can modify the immune response and metabolite levels, leading to constant low-grade inflammation can lead to cartilage injury and frailty [9, 10]. Therefore, the role of the microbiome in cartilage health is now considered important and the study of this new cartilage-gut-microbiome axis will undoubtedly lead to new treatment options for joint diseases such as KBD. A number of studies have shown that many microbial metabolites could affect the development of osteochondral disease [11, 12], suggesting that a diverse gut microbiome could affect an improvement in the metabolic relationship between gut microbes and their hosts [13].

Metabolomics is a relatively new field that studies the signature metabolites expressed by a particular biological system. Metabolites are considered to be the intermediates and end products of cellular biochemical processes, and can be found in various bodily fluids, such as serum, urine, feces, cartilage, and synovial fluid [14]. Their steady-state characteristics can be regarded as the ultimate response of biological systems to the genotype, phenotype, and environment [15]. Gut microbiota translocation could occur in subchondral bone marrow and deeper zones of cartilage, and the complex chemical substances presented by dietary and host nutrients can be converted into metabolites through biochemical converters [16]. These metabolites could play toxicity or injury functions by affecting cartilage metabolism, such as by progressively producing low-grade inflammation in chondrocytes [9]. The transient translocation of the gut microbiota to the subchondral bone marrow/deeper zone of cartilage described above might also contribute to explaining the pathogenesis of KBD. However, the interaction between the gut microbiota and metabolites and their roles in the development of KBD have not yet been effectively reported.

## Results

### Information about the clinical characteristics of the population

The demographic characteristics of the two groups were generally matched, suggesting that none of the established confounding factors influenced group discrimination prior to the experimental design and sample collection. A total of 32 patients with KBD and 35 healthy controls were recruited from Xunyi County, one of the endemic areas for KBD in China; individuals with comparable eating habits were selected to exclude dietary differences (Table S1).

### Alterations in gut microbiota composition in patients with KBD based on 16S rDNA data

In the microbiome study, 5,513,088 high-quality 16S rDNA reads were obtained, with a median of 55,687 reads (range from 35,835 to 91,295) per sample (Supplementary Table S2). A total of 9316 features from 67 samples were generated (Supplementary Table S3), and detailed information about the 16S rDNA data from all samples is provided in Supplementary Table S4.

**Fig. 1:**
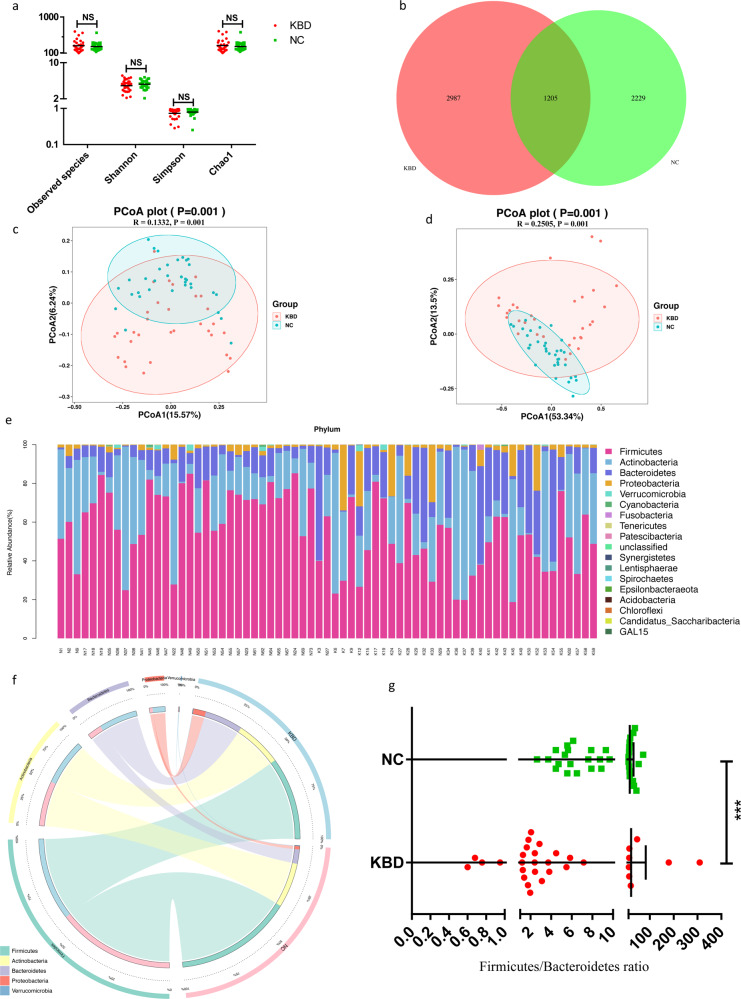
Gut microbiome diversity and structure analysis. **a** Species diversity differences between the KBD and NC groups were estimated by the observed species, Shannon, Simpson, and Chao1 indices. NS, not significant. KBD, patient in the KBD group; and NC, Normal control group. **b** Venn diagram of the observed features in KBD and NC. **c**, **d** Principal coordinate analysis (PCoA) of the microbiota based on the unweighted (ANOSIM, *R* = 0.1332, *P* = 0.001) and weighted (ANOSIM, *R* = 0.2505, *P* = 0.001) UniFrac distance matrices for the KBD and NC groups. **e** Component proportions of bacterial phylum in each group; *n* = 32 for the KBD group and *n* = 35 for the NC group. **f** Circos plot. The left side of the circle represents species and the right side represents sample groups, different colors represent different taxonomic categories and sample groups. From left to right, the thickness of the same color line in the inner ring represents the relative abundance of the species in different sample groups, From right to left, the thickness of the same color line in the inner ring represents the proportion of different species in the sample group. **g** The ratio of Firmicutes/Bacteroidetes of NC showed significantly higher than that of KBD.

To evaluate the characterization of the gut microbiome associated with KBD, alpha diversity and beta diversity between KBD subjects and normal controls were compared. There were no statistically significant differences in the Shannon, observed species, and Chao1 indices (Fig. 1a and Supplementary Table S5). The Venn diagram showed 2987 unique features in the KBD group and 2229 unique features in the normal control (NC) group. A total of 1205 features were shared by both groups (Fig. 1b). The principal coordinates analysis (PCoA) was used to study the extent of the similarity of the microbial communities between the two groups based on unweighted and weighted UniFrac distance metrics (Fig. 1c, d).

### Alterations in the composition of the fecal microflora associated with KBD

The microbial taxon assignment was performed to assess the relative proportions of dominant taxa at the phylum level in both the KBD and NC groups. Considerable variability was observed in the gut microbiota across the samples in each group (Fig. 1e). Eighteen phyla were identified in each group. *Firmicutes* was the most predominant phylum, accounting for 45.88% and 65.06% of the features in the KBD and NC groups, respectively. In addition, *Actinobacteria* (25.66% versus 24.78%) and *Bacteroidetes* (21.18% versus 7.98%) were enriched in the KBD group compared to those in the NC group (Fig. 1f and Supplementary Table S6).

At the phylum level, the KBD group was characterized by higher *Fusobacteria* and *Bacteroidetes* levels (Supplementary Figure S1a) and a significantly higher *Firmicutes/Bacteroidetes* ratio but lower *Firmicutes* levels (Fig. 1g). At the genus level, a total of 56 genera were identified to be significantly differentially abundant between the two groups (Table S7). Of these discriminatory taxa, *Muribaculaceae_unclassified*, *Actinomyces*, *Alloprevotella*, *Fusobacterium*, and *Prevotella_9* were found to be significantly more abundant in the KBD group than in the NC group (Supplementary Figure S1b).

**Fig. 2:**
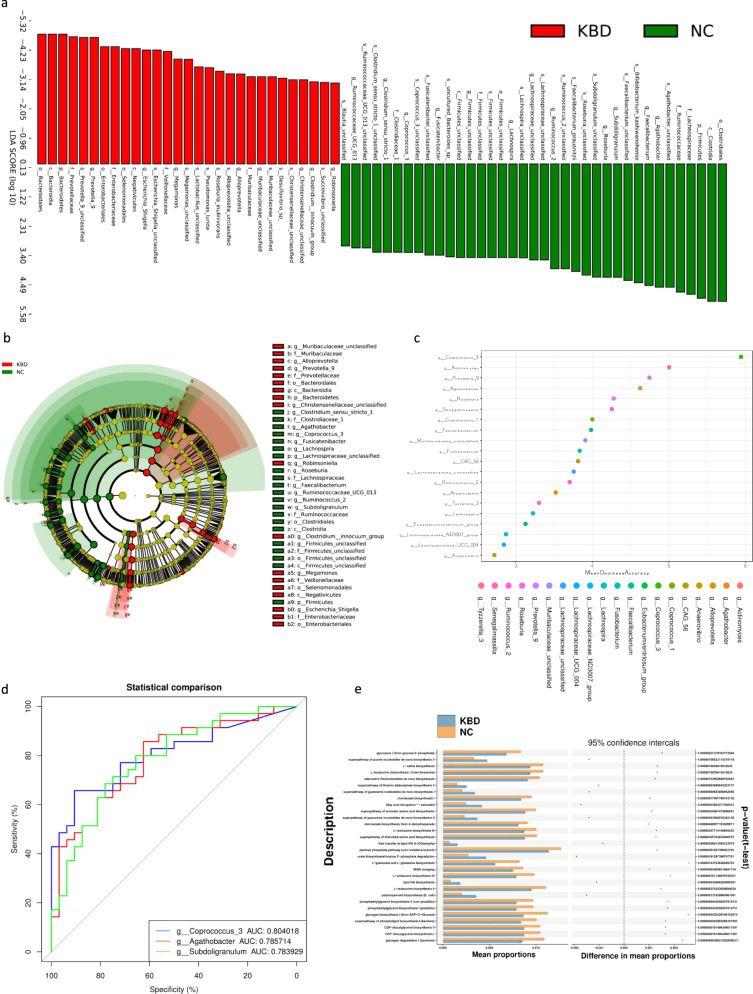
Gut microbiome differential and functional analysis. **a** Linear discriminant analysis (LDA) integrated with effect size (LEfSe). The differences in abundance between the KBD and NC group. **b** Cladogram indicating the phylogenetic distribution of microbiota correlated with the KBD and NC group. **c** Classification performance of a random forest model using 16s rRNA genus abundance assessed by R random Forest package. **d** ROC curve displaying the top 3 biomarkers for classification between KBD and NC. AUC, area under curve. **e** Predicted function of gut microbiota based on KEGG pathway analysis. The extended error bar plot showed the significantly different KEGG pathways between KBD and NC group.

We performed linear discriminant analysis (LDA) integrated with effect size (LEfSe) to generate a cladogram to identify the specific bacteria involved with KBD (Fig. 2b). LDA distribution diagram analysis (LDA score >3) showed a clear change in the microbiota characterized by higher *Bacteroidetes* and *Prevotella 9* levels in the KBD group (Fig. 2a). However, *Firmicutes* levels were significantly decreased in the KBD group (Fig. 2a). The genera *Alloprevotella*, *Robinsoniella*, *Megamonas,* and *Escherichia_Shigella* were more abundant in the KBD group, while the genera *Clostridium_sensu_stricto_1*, *Agathobacter*, *Coprococcus_3*, *Fusicatenibacter*, *Lachnospira*, *Roseburia*, *Faecalibacterium*, *Ruminococcaceae_UCG_013*, *Ruminiococcus_2*, and *Subdoligranulum* were more abundant in the NC group (Fig. 2b).

### The potential role of gut microbiota biomarkers in the risk assessment of KBD

We performed a random forest model based on the differentially abundant genera to detect several potential diagnostic biomarkers that could be used to predict the KBD and NC groups. The optimal model utilized 20 genera and provided the best discriminatory power (Fig. 2c). According to the above analysis, the distribution of the microbial community between KBD and NC subjects showed significant differences. Then, to explore the potential value of the identified bacterial biomarkers for discrimination of the two groups (KBD and NC), we produced receiver operating characteristic (ROC) curves and computed the area under the curve (AUC) values. The top 3 AUC values were for Coprococcus_3 with 80.40%, Agathobacter with 78.57%, and Subdoligranulum with 78.39% (Fig. 2d).

### Prediction of gene function in the gut microbiota

In this study, we used the phylogenetic investigation of communities by reconstruction of unobserved states (PICRUSt2) method to compare gut microbial gene functions across the Clusters of Orthologous Genes (COGs), enzyme nomenclature (EC), Kyoto Encyclopedia of Genes and Genomes (KEGG), KEGG orthology functional orthologues (KO), protein families (PFAM), and protein families featuring curated multiple sequence alignments (TIGRFAM) databases between the KBD and NC groups. Some important functions were identified, such as L-glutamate and L-glutamine biosynthesis and polyisoprenoid biosynthesis among the KEGG pathways (Fig. 2e) and superfamily II DNA and RNA helicase and ADP-glucose pyrophosphorylase in the COG database. The top 30 functions of each database above can be found in Supplementary Figure S2.

### Metagenomic sequencing revealed significant differences among the grade I and II KBD and NC groups

**Fig. 3:**
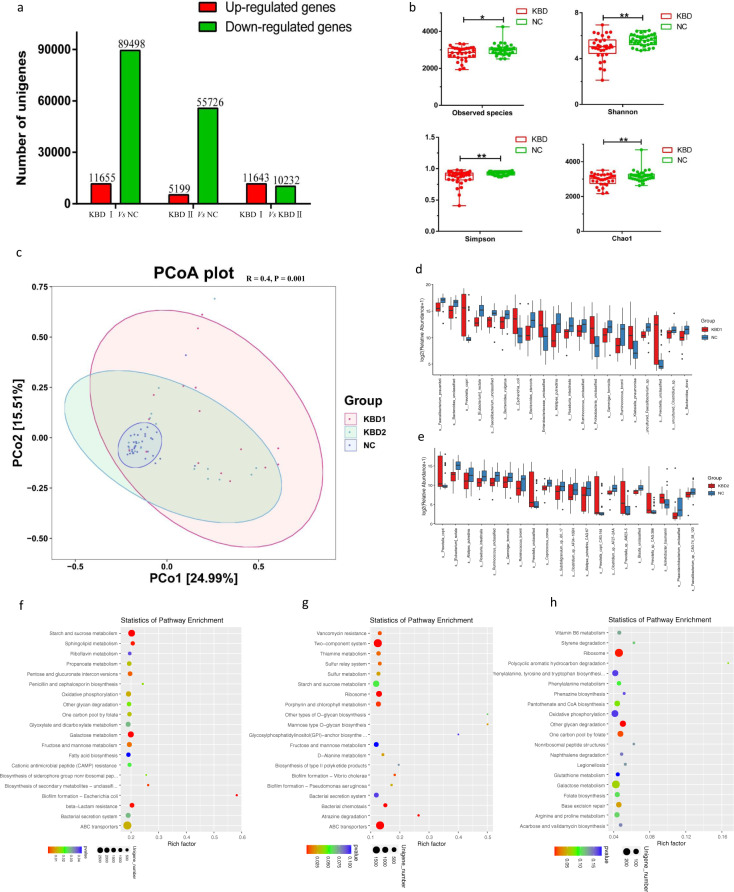
The gut microbiota differences in grade I, II KBD, and NC group based on the metagenomic sequencing data. **a** Differential expressed unigenes among KBD grade I, II, and NC group. **b** Alpha diversity differences between the KBD and NC groups were estimated by the observed species, Shannon, Simpson, and Chao1 indices. *P < 0.05, **P < 0.01. KBD, patient with KBD group; NC, normal control group. **c** The PCoA analysis based on the Bray-Curtis distance matrix between the grade I, II KBD, and NC group at the species level (ANOSIM, *R* = 0.4, *P* = 0.001). **d**, **e** The relative abundance of top 20 species enriched in grade I KBD versus NC. The relative abundance of top 20 species enriched in grade IIvKBD versus NC. The box represents the interquartile ranges, inner line denotes the median. **f**–**h** Pathway classification based on KEGG enrichment analysis of differentially expressed unigenes between grade I KBD and NC (**f**), grade II KBD and NC (**g**), grade I KBD and grade II KBD (**h**). Rich factor, the ratio of the number of DEGs to the number of total genes in this pathway. Genes with mRNA showing at least a twofold change are shown; adjusted *P* < 0.05 for all data selected.

We performed metagenomic sequencing on fecal samples from 16 grade I KBD, 16 grade II KBD, and 35 NC subjects. A total of 578,718 genes were predicted in our study (Supplementary Figure S3a-c). The samples from the grade I KBD group contained 15,467 specific genes, and 9879 specific genes were found in the samples from the KBD grade II group. The samples from the NC group contained 16,069 and 21,657 specific genes compared to grade I and II KBD samples, respectively (Supplementary Figure S3d-f). When compared to those in the NC group, 101,153 differentially expressed unigenes (11,655 upregulated and 89,498 downregulated unigenes) were identified in the grade I KBD group, and 60,925 differentially expressed unigenes (5199 upregulated and 55,726 downregulated unigenes) were identified in the grade II KBD group. A total of 21,875 differentially expressed unigenes (11,643 upregulated and 10,232 downregulated unigenes) were identified between the grade I and II KBD groups (Fig. 3a).

The alpha diversity was significantly lower in the grade I and II KBD groups than in the NC group, as measured by Shannon, observed species, Simpson, and Chao1 indices (Fig. 3b). The PCoA based on the Bray-Curtis distance matrix uncovered striking differences in microbial composition among the grade I and II KBD and NC groups at the species level (Fig. 3c). Then, we compared the profile differences among the grade I and II KBD and NC groups (Supplementary Table S7). The species *Bacteroides vulgatus, Bacteroides stercoris*, and *Bacteroides dorei* had significantly decreased abundance in the grade I KBD group compared to those in the NC group (Fig. 3d, Table S7), *Prevotella copri, Prevotella copri-CAG:164, Prevotella SP-AM23-5,* and *Prevotella SP-CAG:386* had significantly increased abundance in the grade II KBD group compared to those in the NC group (Fig. 3e, Supplementary Table S8), whereas when comparing species between grade I and II KBD, 6 species from *Bacteroides* had decreased abundance in the grade I KBD group, 6 *Collinsella* species had decreased abundance in the grade II KBD group (Supplementary Figure S3g, Supplementary Table S8).

### Functional analysis of metagenomic sequencing in the grade I and II KBD groups

We selected the top 10 GO items of the three forms of definitions by the GO database. Results of GO function classification analysis of differentially expressed unigenes between the different groups are shown in Supplementary Figure S4a-c. GO enrichment analysis of differentially expressed unigenes in different groups was performed, and the top 20 GO terms can be found in Supplementary Figure S5d-f. Finally, KEGG analysis of differentially expressed unigenes showed the most abundant metabolic pathway in different groups. Starch and sucrose metabolism, galactose metabolism and sphingolipid metabolism differed between the grade I KBD and NC groups (Fig. 3f and Supplementary Table S9); ATP-binding cassette (ABC) transporters, two-component system, and bacterial chemotaxis differed between the grade II KBD and NC groups (Fig. 3g and Supplementary Table S9); and ribosome, glutathione metabolism, and vitamin B6 metabolism differed between the grade I KBD and grade II KBD groups (Fig. 3h and Supplementary Table S9). The analysis further suggested that these disrupted pathways in different groups might play a key role in the potential interaction effects between KBD and cartilage injuries generated by the dysbiosis of the gut microbiome and serum metabolites.

### Metabolomic analysis revealed abnormal metabolic alterations in patients with grade I and II KBD

**Fig. 4:**
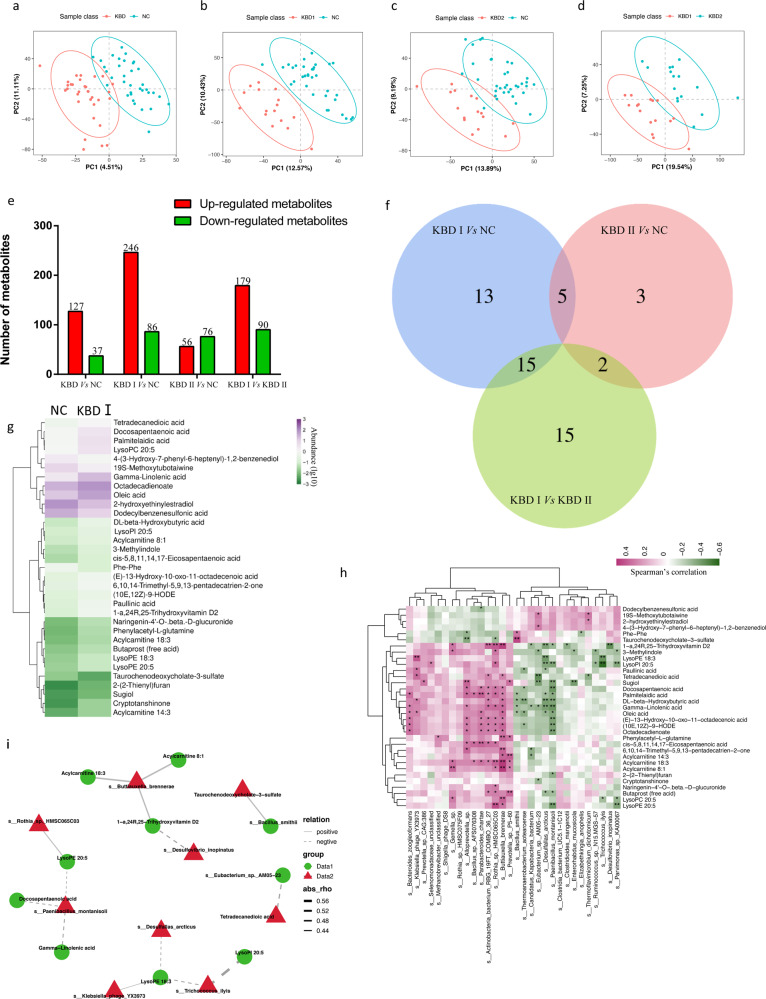
The aberrant metabolic patterns in KBD (grade I and II) compared with normal controls. **a**–**d** The clustering analyses of partial least-square discriminant analysis (PLS-DA). **e** Differential regulated metabolites among KBD grade I, II and NC group. **f** Venn diagram displaying the common significantly different metabolites among three groups. **g** Heat map of the 32 idms2 significantly different metabolites across grade I KBD and normal controls. Metabolites >2-fold changes, VIP ≥ 1, *P* < .05 (T test). **h** Correlations between species and metabolites. The top 30 species were detected in Metagenomic data. Metabolites >2-fold changes between grade I KBD and normal controls, with *P* < 0.05 (T test), VIP ≥ 1. The correlation effect is indicated by a color gradient from green (negative correlation) to red (positive correlation). *P < 0.05, **P < 0.01, T test. **i** The correlation network of significantly different metabolites and species across grade I KBD and normal controls, abs_rho means abundances correlation coefficient.

Partial least squares discriminant analysis (PLS-DA) was performed to determine the profile of discriminant metabolites of serum samples from distinct groups, indicating a good ability to classify individuals according to their KBD status (Fig. 4a–d). We identified 178 differentially regulated metabolites (DRMs) between the KBD and NC groups, 285 DRMs between the grade I KBD and NC groups, 191 DRMs between the grade II KBD and NC groups, and 305 DRMs between the grade I KBD and grade II KBD groups (Fig. 4e). The common DRMs among the three groups are shown in Fig. 4f. Gamma-linolenic acid, palmitelaidic acid, 3-oxoctadecanoic acid, and oleic acid were more abundant, while taurochenodeoxycholate-3-sulfate and Phe-Phe were less abundant in grade I KBD and grade II KBD samples than in control samples (Fig. 4g, Supplementary Figure S5a, b). The DRMs were mapped to differentially abundant KEGG metabolic pathways, including biosynthesis of unsaturated fatty acids (4 metabolites), glycerophospholipid metabolism (3), and linoleic acid metabolism (2) (Supplementary Table S10). According to DRMs and differential KEGG metabolic pathways noted above, the metabolism of lipids and lipid-like molecules was the most significantly different in this study, which suggested that a disturbance of lipids might occur in KBD patients.

A correlation matrix was created using Spearman correlation and a correlation network analysis to explore the potential relationships between changes in the gut microbiome and metabolic products (Fig. 4h, i, Supplementary Figure S5c, d). In the grade I KBD and NC groups, the levels of unsaturated fatty acids, such as docosapentaenoic acid, palmitelaidic acid, gamma-linolenic acid, oleic acid, and (E)-13-hydroxy-10-oxo-11-octadecenoic acid, were negatively correlated with the abundance of *Paenibacillus montanisoli* and positively correlated with the abundance of *Buttiauxella brennerae*. The levels of lysolecithins, such as LysoPE 20:5, LysoPC 20:5, and LysoPI 20:5, were negatively correlated with the abundance of the species *Parvimonas sp. KA00067* and positively correlated with the abundance of the species *Rothia sp. HMSC065C03* (Fig. 4h, i). In the grade I KBD and grade II KBD groups, the levels of acylcarnitine and lysolecithins were positively correlated with the abundance of the species *Prevotella sp. P5-60* and *Rothia sp. HMSC065C03* and negatively correlated with the abundance of the species *Bacteroides sp. AM25-34* and *Parabacteroides sp. AF18-52* (Supplementary Figure S5c, d and Supplementary Figure S5e, f).

## Discussion

The gut microbiota is located in the entire gastrointestinal tract, exists in a dynamic state and reflects a real ecosystem. More than 10^14^ bacteria and more than 1000 bacterial species as well as fungi, viruses, phages, parasites, and archaea comprise the whole gut microbiota [17]. *Bacteroidetes* and *Firmicutes* are the most typical bacterial phyla of a healthy gut microbiota, followed by *Actinobacteria*, *Fusobacteria*, and *Proteobacteria*, and the most typical genera are *Bacteroides*, *Faecalibacterium*, and *Bifidobacterium* [18]. However, Rogier et al. [19] found a decrease in *Bacteroidaceae* abundance and an increase in *Firmicutes* abundance in a collagen-induced arthritis (CIA) mouse model. In a previous study by Scher et al. [20], the patients with new-onset RA had a lack of gut *Bacteroides* when compared with healthy individuals. In our study, we found an alteration of the microbiota characterized by elevated *Bacteroidetes* and lower *Firmicutes* levels in the KBD group, which indicates dysbiosis of the healthy gut microbiota in KBD patients. In addition, the anti-inflammatory species *Faecalibacterium prauznitzii* showed a consistent depletion in grade I KBD in this study, which is consistent with direct correlation research between ageing and gut dysbiosis [21]. Cindy G. Boer et al demonstrated a relationship between a greater abundance of *Streptococcus spp*. and higher osteoarthritis-related knee pain [22]. *Streptococcus spp*. were also present at higher levels in KBD patients than in NC subjects. *Streptococcus spp*. might induce or exacerbate joint inflammation, and the precise mechanisms of joint inflammation triggered by *Streptococcus spp*. may involve metabolites produced by *Streptococcus spp*. in the gastrointestinal tract [22].

*Bacteroides* accounts for more than 10% of the total human gut microbiota and is mainly responsible for degrading polysaccharides and dietary fibers [23]. Chondroitin sulfate degradation by *Bacteroides* strains isolated from the human gut microbiota has been studied by Salyers et al. [24]. *Prevotella* is one of the most dominant bacteria in the human intestinal tract [25, 26]. Recently, a study showed that *Prevotella copri* can produce succinic acid with utilization of polysaccharides [27], and succinic acid has been considered to enhance the immune response to protect host health [28]. Increasing evidence has shown that many KBD patients display immune dysfunction [29–31]. Therefore, it can be speculated that their immune disorder may be related to the depletion of *Prevotella* bacteria in the intestine; however, *Prevotella spp*. were found to be significantly enriched in grade II KBD, which may be due to the long-term oral administration of chondroitin sulfate and other nutrients in KBD patients.

Diet is one of the important environmental factors leading to inter-individual differences in physiologic gut microbiota composition [32, 33]. Nutrients are essential not only for human health, but for the normal homeostasis of the trillions of microbes in the gut microbiota [34]. For decades, selenium deficiency and *Fusarium* mycotoxins (T-2 toxin, deoxynivalenol, and nivalenol) were found to be two of the main risk factors for KBD [35, 36]. Kasaikina et al. suggested that selenium could cause unique effects across microbial taxa by increasing the diversity of the microbiota in mice [37]. Probiotics seem to have the ability to accumulate selenium and incorporate it into organic compounds [38]. Some researchers demonstrated that there could be a synergistic effect for health between selenium and probiotics via different pathways during incorporation, especially in deficient conditions [37, 39]. In addition, administration of selenium nanoparticles was demonstrated to improve gut health by increasing the abundance of beneficial bacteria, such as *Lactobacillus* and *Faecalibacterium* [40]. Therefore, the selenium deficiency status in vivo caused by low selenium in the external environment may crucially affect the composition of the intestinal flora in KBD patients.

Silvia W. Gratz et al. found that deoxynivalenol-Glc, nivalenol-Glc, and T-2 toxin-Glc were stable in gastrointestinal digestive juices and were not efficiently transported through intestinal epithelial cell monolayers. However, upon contact with the human gut microbiota, they were hydrolyzed efficiently [41]. Therefore, the abnormal alteration of the gut microbiota in KBD could affect the hydrolysis efficiency, which ultimately causes masked *Fusarium* mycotoxins to be released as parent mycotoxins and enter systemic circulation through intestinal epithelial cells. We infer that the imbalance of dietary nutrients and the low-selenium diet with *Fusarium* mycotoxin contamination could be one of the most important factors affecting the gut microbiota composition in KBD patients, which leads to joint degradation through the cartilage-gut-microbiome axis.

The metabolome was defined as the small molecules released by cells and provided a path for investigating how mechanistic biochemistry evolves into cellular phenotypes [42]. Genes and proteins often have functions in epigenetic regulation and post-translational modifications, respectively. However, metabolites play a direct role in signatures of biochemical activity and therefore tend to reflect the relationship with the phenotype [43]. Metabolomics has produced a relatively large number of small-molecule metabolites from body fluids or tissues, which have been detected and measured quantitatively in a single step. In addition, differential visual data and vivid figures promise immense potential for understanding the pathogenesis of many diseases [44]. Increasing evidence has suggested unfavorable effects of fatty acids, with increased production of pro-inflammatory and pro-apoptotic markers. Several studies have found that palmitoleic acid could induce greater cartilage degeneration and increase MMP-13 and collagenase 10 expression in a mouse study [45, 46]. Several studies have recognized the potential role of glycerophospholipids (including phosphatidylcholines (PCs) and lysoPCs) in the synovial fluid and in the serum of OA subjects [47–51]. In this study, our results showed altered levels of several PCs, lysoPCs and sphingomyelins (SMs) in grade I KBD and grade II KBD. Glycerophospholipids can form the lipid bilayer and be involved in cell signaling and the regulation of membrane transport [52]. Specifically, PCs and SMs constitute over 50% of the cell membrane [14]. Phospholipids are metabolized by phospholipase A1 and A2, which hydrolyze the ester bonds of fatty acid chains related to the glycerol backbone. The elimination of the fatty acid chain leads to lysoPC formation. Phospholipids, one of the three important components of synovial fluid, are responsible for transporting oxygen and nutrients to the cartilage [53]. Dysregulation of lipid metabolism has been reported in OA joints and is considered as an important pathophysiological feature of the disease [48, 54, 55]. LysoPCs are crucial components of oxidized low-density lipoprotein (LDL) cholesterol (oxLDL), the levels of which have been found to correlate with OA severity [56] and with KBD severity. More LysoPCs were identified as DRMs in grade II KBD than in grade I KBD. In addition, correlation network analysis suggested that gut microbiota dysbiosis is closely correlated with KBD development and that lipid metabolism dysregulation might be a crucial factor.

The sample size is a main flaw of this study. First, the gradually decreased incidence of KBD and elderly cases have died gradually in the past decade, which caused the number of patients with KBD has dropped dramatically. Second, the pathological characteristics of some adult KBD have been changed or merged with OA, making the collection of a typical KBD case extremely complex. We establish extremely strict inclusion criteria in this study. Therefore, it is very difficult to collect a large number of biological sample from patients with KBD by the two limitations above. In this study, we selected 32 patients with KBD and 35 healthy controls, which is a relatively small sample size. However, the extremely strict inclusion criteria could avoid the statistical bias caused by confounding factors.

**Fig. 5:**
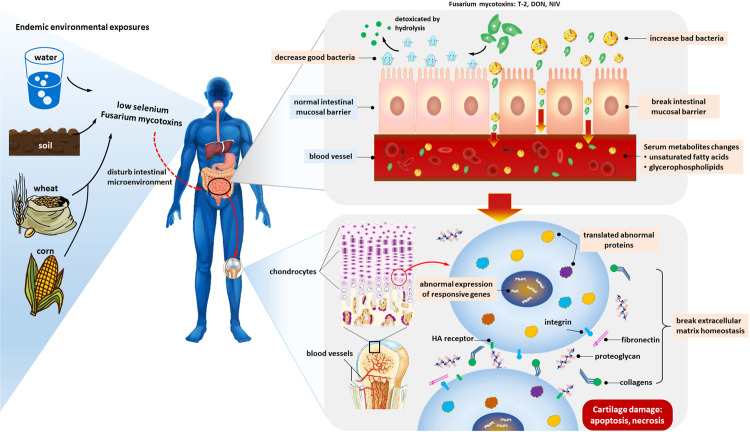
Proposed hypothetical pathophysiological mechanism (environment–gene interaction) explaining the association among the gut microbiota, serum metabolites, environmental risk factors, and cartilage injury. No causality has been established between the gut microbiota and KBD-related cartilage injury; however, if such causality exists, we propose the following model. First, the selenium deficiency status in vivo caused by low-selenium levels in the external environment affects the composition of the intestinal flora in KBD patients, and the endemic environmental risk factors, masked *Fusarium* mycotoxins, enter the human body. Due to abnormal alteration of the gut microbiota caused by selenium deficiency, the efficiency of the hydrolysis of masked *Fusarium* mycotoxins by the intestinal tract is abnormal, which causes masked Fusarium mycotoxins to be released as parent mycotoxins and harmful bacteria entering the systemic circulation, causing abnormal lipid metabolism. Then, translocated *Fusarium* mycotoxins and harmful bacteria from the intestinal tract to the subchondral bone marrow enter the deep zone of articular cartilage and hypertrophic layers of epiphyseal plate cartilage, which are the primary lesion sites of KBD. Finally, the responsive genes of chondrocytes are triggered by the risk factors noted above, which can directly contribute to KBD cartilage damage, such as apoptosis and necrosis.

In conclusion, our study revealed that KBD patients showed gut dysbiosis at the phylum, genus, and species levels. The changed species associated with metabolite alterations in grades I and II KBD were identified. In particular, interaction analysis among the gut microbiota, metabolites, and cartilage loss was discussed, which could provide clues for better understanding the mechanisms underlying the pathogenesis of KBD and potentially reveal whether the origin of such alterations in epiphyseal plate cartilage can be linked to the gut microbiota. Based on the cartilage-gut-microbiome axis, we have summarized this hypothetical model in Fig. 5, involving the gut microbiota, serum metabolites, environmental risk factors, and cartilage injury, and may provide a new perspective in terms of KBD pathogenesis, which better integrates environmental risk factors with cartilage damage in KBD.

## Materials and methods

### Study design and sample collection

A total of 46 patients with KBD and 57 healthy controls from Xunyi county, one of the endemic areas for KBD in China, were recruited for this study. The patients with KBD were diagnosed strictly according to the national diagnostic criteria of KBD in China [WS/T 207-2010]. All subjects were diagnosed with KBD when manifested with X-ray alterations, such as defects and sclerosis on the bone end of phalanges combined with compression changes of the calcaneus and talus, and enlarged/deformed limb joints (hand, elbow, knee, ankle, etc.). Subjects were excluded for the following reasons: they were suffering or had previously suffered from any other osteoarticular diseases (such as osteoarthritis, rheumatoid arthritis, gout, or skeletal fluorosis) or any other type of macrosomia, osteochondrodysplasia, or chronic disease (such as hypertension, diabetes, or coronary heart disease), they had accepted any treatment in the past 6 months, or they had a history of inflammatory bowel disease (IBD), irritable bowel syndrome (IBS), or complications of complete intestinal obstruction. All the healthy controls did not have any musculoskeletal pathologies or recent injuries, had a normal bowel habit and had no history of IBS, IBD, CRC, or other severe gastroenterological disease. All patients and healthy controls using antibiotics, probiotics, prebiotics, or synbiotics within two months of sampling were excluded. All subjects signed a written informed consent form. Their general clinical data including age, sex, educational background, and body mass index (BMI), were recorded. All patients and healthy controls were Shaanxi Han Chinese from similar geographic areas with similar eating habits. According to the inclusion and exclusion criteria listed above, we finally selected 32 patients with KBD and 35 healthy controls in this study. All qualified stool samples were self-sampled prior to mechanical feces preparation and were transported immediately to the laboratory, divided into three portions per sample, packed into three freezer tubes, frozen in liquid nitrogen overnight, and preserved at −80 °C for further testing.

### DNA extractions and 16S rDNA gene sequencing

DNA from different samples was extracted using the E.Z.N.A.® Stool DNA Kit (D4015-02, Omega, Inc., USA) according to the manufacturer’s instructions. The reagent that was designed to uncover DNA from trace amounts of sample has been shown to be effective for the preparation of DNA from most bacteria. Sample blanks consisted of unused swabs processed through DNA extraction and tested to contain no DNA amplicons. The total DNA was eluted in 50 µl of elution buffer by a modification of the procedure described by the manufacturer (QIAGEN) and stored at −80 °C until measurement by PCR at LC-BIO TECHNOLOGIES (HANGZHOU) CO., LTD., Hang Zhou, Zhejiang Province, China.

The V3-V4 region of the prokaryotic (bacterial and archaeal) small-subunit (16S) rDNA gene was amplified with the primers 341F (5′-CCTACGGGNGGCWGCAG-3′) and 805R (5′-GACTACHVGGGTATCTAATCC-3′). The 5′ ends of the primers were tagged with specific barcodes for each sample and were sequenced with universal primers. PCR amplification was performed in a 25 μL reaction mixture containing 25 ng of template DNA, 12.5 μL of PCR Premix, 2.5 μL of each primer, and PCR-grade water to adjust the volume. The PCR conditions to amplify the prokaryotic 16S fragments consisted of an initial denaturation at 98 °C for 30 s; 32 cycles of denaturation at 98 °C for 10 s, annealing at 54 °C for 30 s, and extension at 72 °C for 45 s; and a final extension at 72 °C for 10 min. The PCR products were confirmed with 2% agarose gel electrophoresis. Throughout the DNA extraction process, ultrapure water, instead of sample solution, was used to exclude the possibility of false-positive PCR results as a negative control. The PCR products were purified by AMPure XT beads (Beckman Coulter Genomics, Danvers, MA, USA) and quantified by Qubit (Invitrogen, USA). The amplicon pools were prepared for sequencing and the size and quantity of the amplicon library were assessed on an Agilent 2100 Bioanalyzer (Agilent, USA) and with the Library Quantification Kit for Illumina (Kapa Biosciences, Woburn, MA, USA), respectively. The libraries were sequenced on the NovaSeq PE250 platform.

According to the manufacturer’s recommendations(LC-Bio). Paired-end reads were assigned to samples based on their unique barcode and truncated by cutting off the barcode and primer sequence. Paired-end reads were merged using FLASH. Quality filtering on the raw reads was performed under specific filtering conditions to obtain the high-quality clean tags according to fqtrim (v0.94). Chimeric sequences were filtered using Vsearch software (v2.3.4). After dereplication using DADA2, we obtained a feature table and feature sequence. Alpha diversity and beta diversity were calculated by QIIME2, for which the same number of sequences was extracted randomly by reducing the number of sequences to the minimum for some samples, and the relative abundance was used to determine the bacterial taxonomy. Alpha diversity and beta diversity were analyzed by QIIME2, and figures were drawn in R (v3.5.2). The sequence alignment based on the species annotation was performed by Blast, and the alignment databases used were SILVA and NT-16S.

### Metagenomic sequencing

Sixteen grade I KBD samples, 16 grade II KBD samples, and 35 NCs were used to perform metagenomics analysis. The DNA library was constructed with the TruSeq Nano DNA LT Library Preparation Kit (FC-121-4001). The DNA was fragmented by dsDNA Fragmentase (NEB, M0348S) and incubated at 37 °C for 30 min. Library construction began with fragmented cDNA. Blunt-end DNA fragments were generated using a combination of fill-in reactions and exonuclease activity, and size selection was performed with the sample purification beads provided by the kit. An A-base was then added to the blunt ends of each strand, preparing them for ligation to the indexed adapters. Each adapter contains a T-base overhang for ligating the adapter to the A-tailed fragmented DNA. These adapters contain the full complement of sequencing primer hybridization sites for single, paired-end, and indexed reads. Single- or dual index adapters were ligated to the fragments and the ligated products were amplified with PCR with the following conditions: initial denaturation at 95 °C for 3 min; 8 cycles of denaturation at 98 °C for 15 s, annealing at 60 °C for 15 s, and extension at 72 °C for 30 s; and a final extension at 72 °C for 5 min.

Raw sequencing reads were processed to obtain valid reads for further analysis. First, sequencing adapters were removed from sequencing reads using cutadapt v1.9. Second, low-quality reads were trimmed by fqtrim v0.94 using a sliding-window algorithm. Third, reads were aligned to the host genome using bowtie2 v2.2.0 to remove host contamination. Once quality-filtered reads were obtained, they were de novo assembled to construct the metagenome for each sample by IDBA-UD v1.1.1. All coding sequences (CDS) of in the metagenomic contigs were predicted by MetaGeneMark v3.26. The CDS of all samples were clustered by CD-HIT v4.6.1 to obtain unigenes. Unigene abundance for a certain sample was estimated by TPM based on the number of aligned reads in bowtie2 v2.2.0. The lowest common ancestor taxonomy of unigenes was obtained by aligning them against the NCBI NR database with DIAMOND v 0.9.14. Similarly, the functional annotations (GO, KEGG, eggnog, CAZy, CARD, PHI, MGEs, VFDB) of unigenes were obtained. Based on the taxonomic and functional annotation of unigenes, along with their abundance profiles, the differential analysis was carried out at each taxonomic, functional or gene-wise level by Fisher’s exact test.

### Metabonomic analysis based on liquid chromatography-mass spectrometry (LC/MS)

#### Serum collection and preparation

Approximately 5–7 ml of venous blood was collected from each subject, and rested for 5–10 min. First, the samples were centrifuged at 1500 × *g* for 5 min and the supernatants were collected. Then, the samples were centrifuged at 12,000 × *g* for 10 min, and the supernatant was collected.The resulting serum was then frozen in liquid nitrogen overnight and preserved under −80 °C before testing. None of the samples was thawed more than twice before being analyzed. Our studies were conducted under the institutional review board (IRB) protocols of the participating institutions.

#### LC–MS analysis

All samples were acquired by the LC–MS system following the technical specifications. First, all chromatographic separations were performed using an ultra-performance liquid chromatography (UPLC) system (SCIEX, UK). An ACQUITY UPLC BEH Amide column (100 mm × 2.1 mm, 1.7 µm, Waters, UK) was used for the reversed-phase separation. The column oven was maintained at 35 °C. The flow rate was 0.4 ml/min and the mobile phase consisted of solvent A (25 mM ammonium acetate + 25 mM NH_4_H_2_O) and solvent B (IPA: ACN = 9:1 + 0.1% formic acid). The gradient elution conditions were set as follows: 0–0.5 min, 95% B; 0.5–9.5 min, 95–65% B; 9.5–10.5 min, 65–40% B; 10.5–12 min, 40% B; 12–12.2 min, 40–95% B; 12.2–15 min, 95% B. The injection volume for each sample was 4 µl.

A high-resolution tandem mass spectrometer (TripleTOF5600plus; SCIEX, UK) was used to detect metabolites eluted from the column. The Q-TOF was operated in both positive and negative ion modes. The curtain gas was set to 30 PSI, the ion source gas1 was set to 60 PSI, the ion source gas 2 was set to 60 PSI, and the interface heater temperature was 650 °C. For positive ion mode, the ionspray voltage floats were set to 5000 V. For negative ion mode, the ionspray voltage floats were set to −4500 V. The mass spectrometry data were acquired in IDA mode. The TOF mass range was from 60 to 1200 Da. The survey scans were acquired in 150 ms and as many as 12 product ion scans were collected if they exceeded a threshold of 100 counts per second (counts/s) with a 1+ charge-state. The total cycle time was fixed to 0.56 s. Four time bins were summed for each scan at a pulse frequency value of 11 kHz through monitoring of the 40 GHz multichannel TDC detector with four-anode/channel detection. Dynamic exclusion was set to 4 s. During the acquisition, the mass accuracy was calibrated every 20 samples. Furthermore, in order to evaluate the stability of LC–MS during the whole acquisition, a quality control sample (pool of all samples) was acquired after every 10 samples.

#### Data processing

The acquired MS data pretreatments including peak picking, peak grouping, retention time correction, second peak grouping, and annotation of isotopes and adducts were performed using XCMS software. LC–MS raw data files were converted into mzXML format and then processed by the XCMS, CAMERA, and metaX toolbox implemented in R software. Each ion was identified by combining retention time (RT) and *m*/*z* data. The intensities of each peaks were recorded, and a three-dimensional matrix containing arbitrarily assigned peak indices (retention time–*m*/*z* pairs), sample names (observations) and ion intensity information (variables) was generated.

The online KEGG and HMDB databases were used to annotate the metabolites by matching the exact molecular mass data (*m*/*z*) of samples with those from the database. If the mass difference between the observed value and the database value was <10 ppm, the metabolite would be annotated and the molecular formula of the metabolite would be identified and validated by the isotopic distribution measurements. We also used an in-house fragment spectrum library of metabolites to validate the metabolite identification.

The intensity of the peak data was further pre-processed by an in-house software program metaX. Those features that were detected in <50% of the QC samples or 80% of the biological samples were removed, and the remaining peaks with missing values were imputed with the k-nearest neighbor algorithm to further improve the data quality. PCA was performed for outlier detection and batch effects evaluation using the pre-processed dataset. Quality control-based robust LOESS signal correction was fitted to the QC data with respect to the order of injection to minimize signal intensity drift over time. In addition, the relative standard deviations of the metabolic features were calculated across all QC samples, and those >30% were then removed.

Student *t*‐tests were conducted to detect differences in metabolite concentrations between two phenotypes. The *P* value was adjusted for multiple tests using an FDR (Benjamini–Hochberg). Supervised PLS‐DA was conducted through metaX to discriminate the different variables between groups. The VIP value was calculated, and a VIP cut‐off value of 1.0 was used to select important features.

### Statistical analyses

Significant differences in clinical characteristics were evaluated with Pearson’s chi-square test or Fisher’s exact test. Spearman’s correlation analysis was conducted to calculate the correlation between species and metabolites. Differences were considered significant when *p* < 0.05. All data were analyzed with GraphPad Prism 6 software (GraphPad Software, Inc., San Diego, California, USA), R version 3.5.2 (R Foundation for Statistical Computing, Vienna, Austria), and Microsoft Excel (Microsoft Corporation, Seattle, WA, USA).

## Supplementary information

supplementary figure 1

supplementary figure 2

supplementary figure 3

supplementary figure 4

supplementary figure 5

supplementary Figure legends

Table S1

Table S2

Table S3

Table S4

Table S5

Table S6

Table S7

Table S8

Table S9

Table S10

## Data Availability

The datasets used and/or analyzed during the current study are available from the corresponding author on reasonable request.
