# Supplementary figures and images for: Alterations in the gut microbiota and metabolite profiles of patients with Kashin-Beck disease, an endemic osteoarthritis in China
Source: Cell Death Dis. 2021 Oct 28;12(11):1015. doi: 10.1038/s41419-021-04322-2 (PMC8553765; doi:10.1038/s41419-021-04322-2)

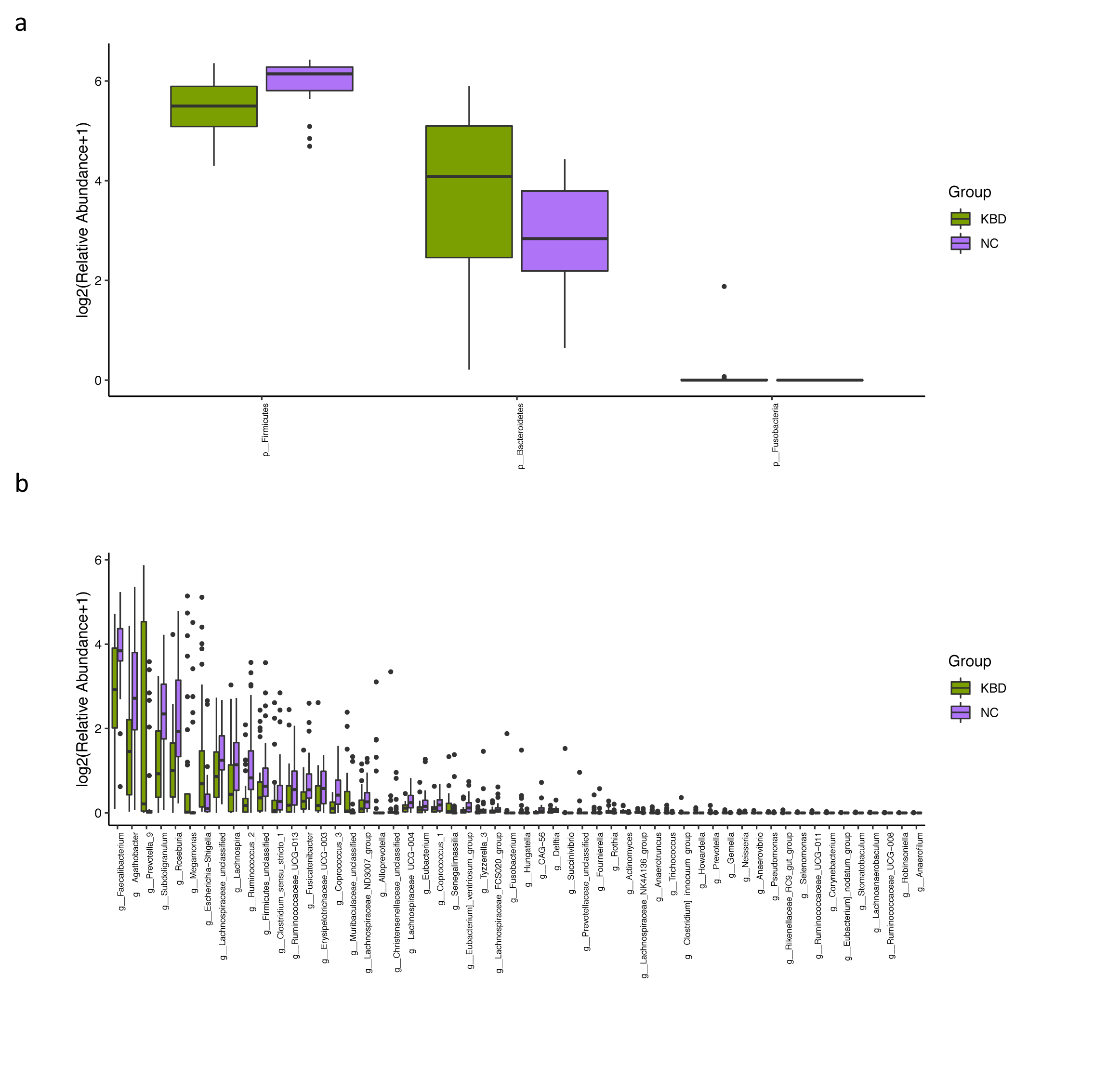

Supplement: Supplementary file 1 — supplementary figure 1 [file 41419_2021_4322_MOESM1_ESM.tif]

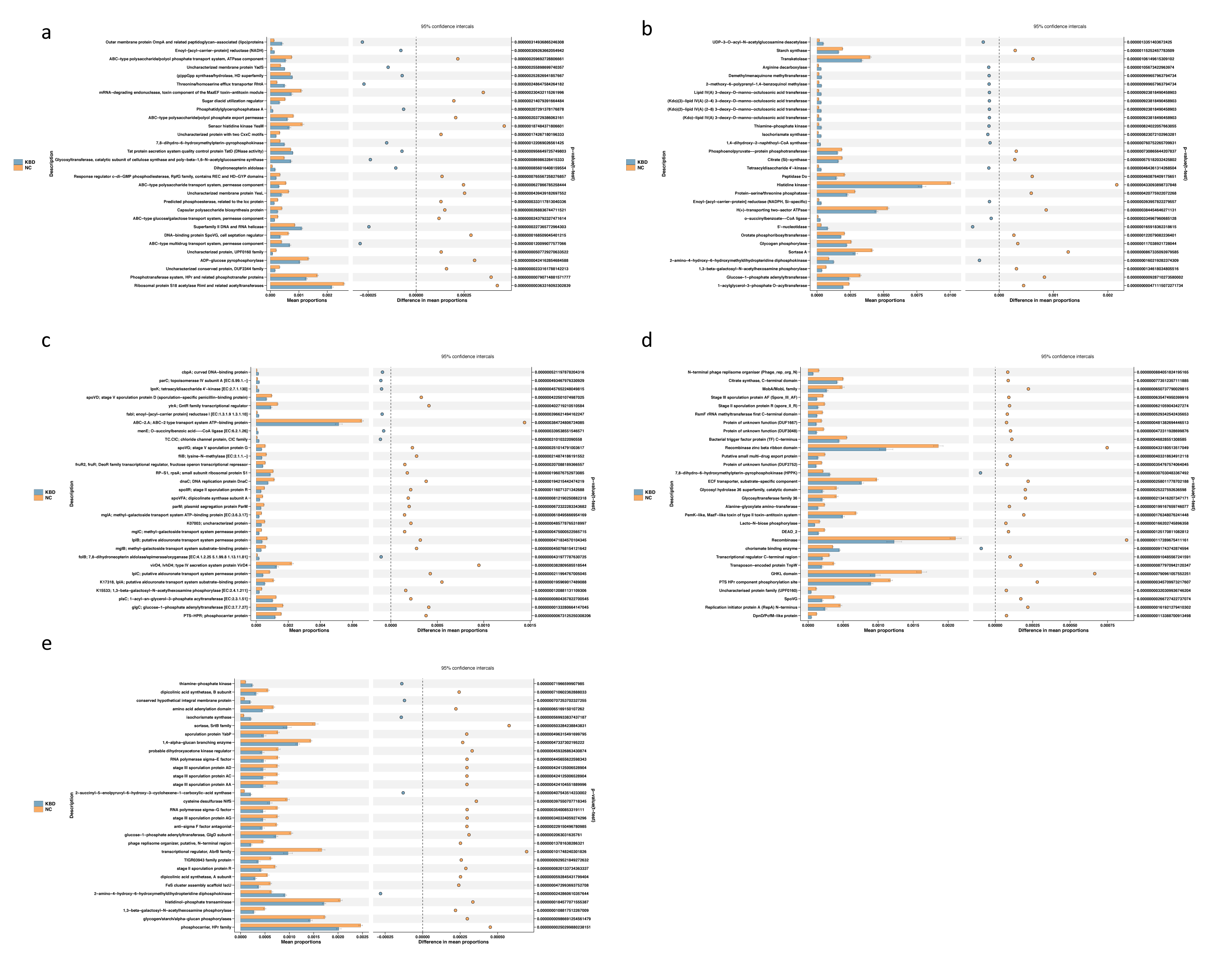

Supplement: Supplementary file 2 — supplementary figure 2 [file 41419_2021_4322_MOESM2_ESM.tif]

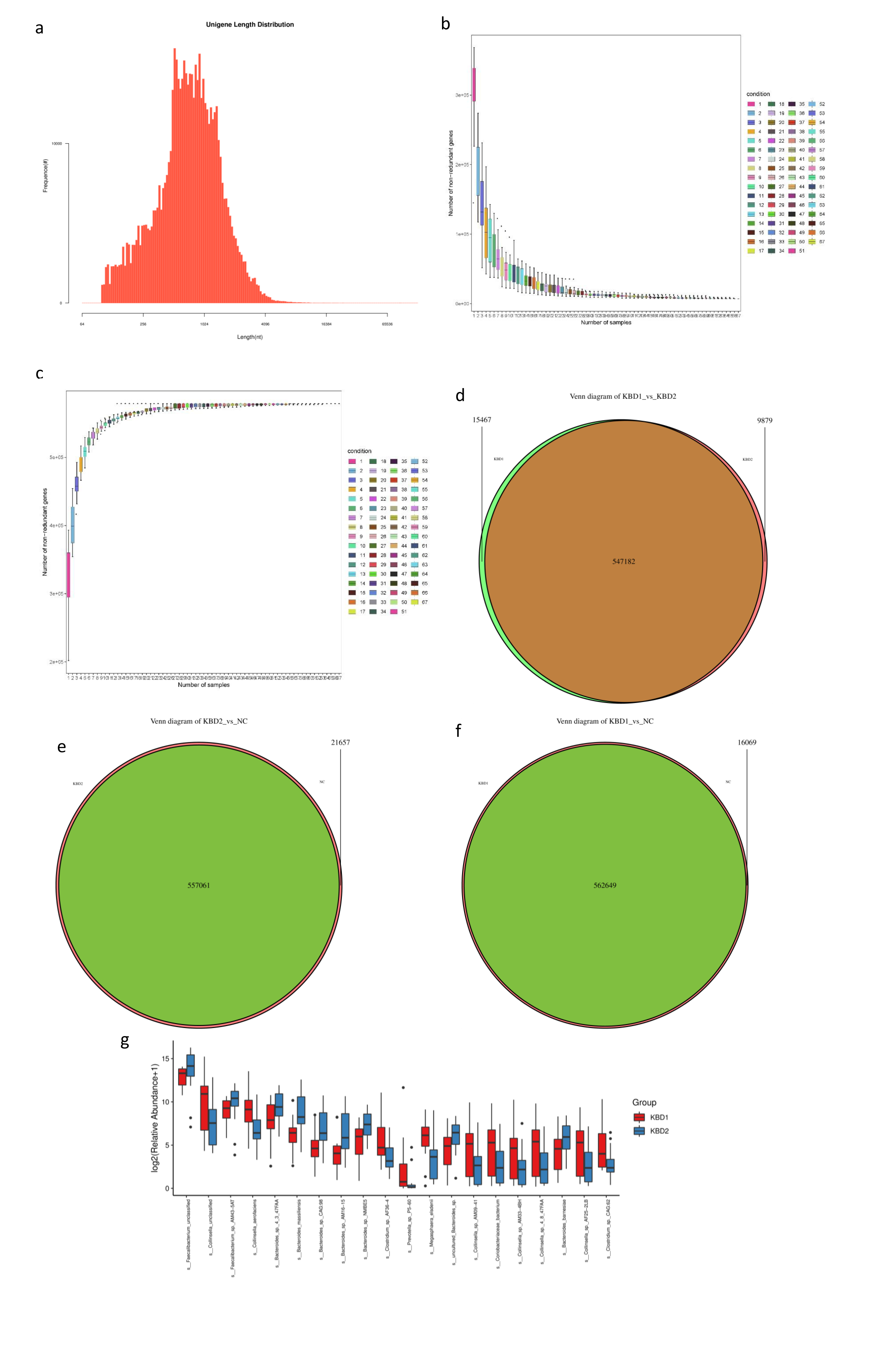

Supplement: Supplementary file 3 — supplementary figure 3 [file 41419_2021_4322_MOESM3_ESM.tif]

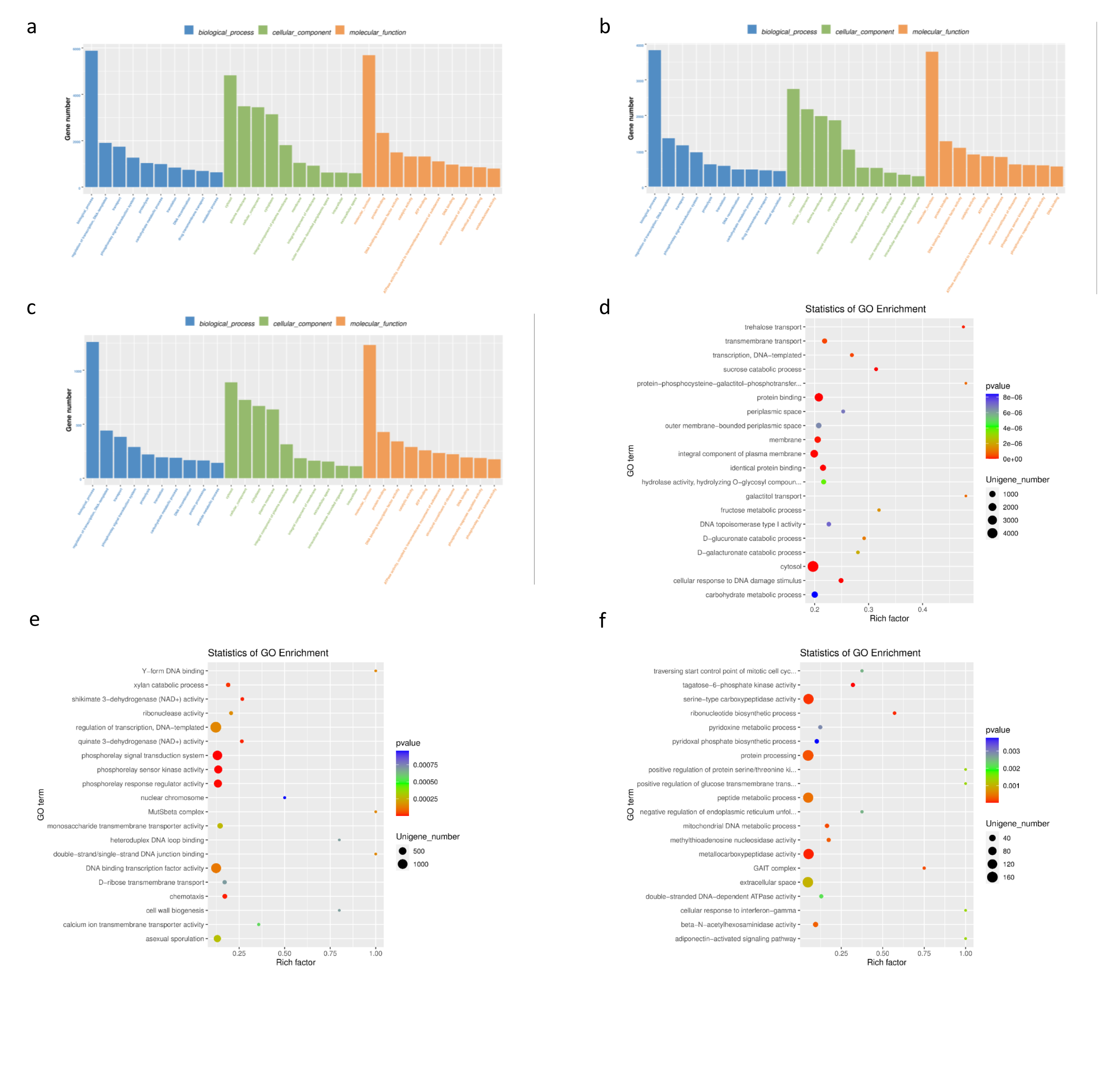

Supplement: Supplementary file 4 — supplementary figure 4 [file 41419_2021_4322_MOESM4_ESM.tif]

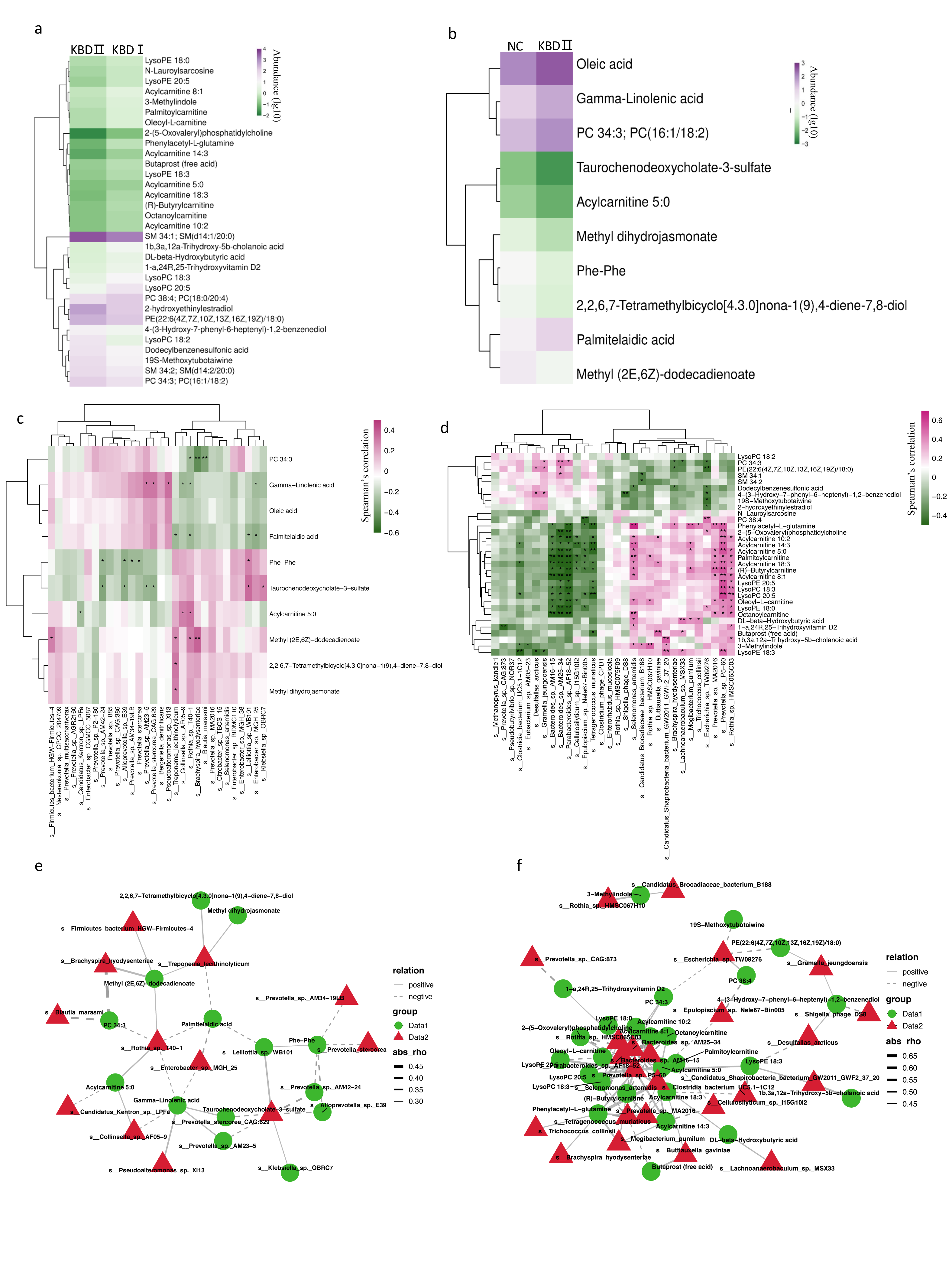

Supplement: Supplementary file 5 — supplementary figure 5 [file 41419_2021_4322_MOESM5_ESM.tif]
