# Supplementary material for: Alterations in the gut microbiota and metabolite profiles of patients with Kashin-Beck disease, an endemic osteoarthritis in China
Source: Cell Death Dis. 2021 Oct 28;12(11):1015. doi: 10.1038/s41419-021-04322-2 (PMC8553765; doi:10.1038/s41419-021-04322-2)
Supplement: Supplementary file 6 — supplementary Figure legends [file 41419_2021_4322_MOESM6_ESM.docx]

**Online Figure legends**

**Figure S1** Alterations in the composition of fecal microflora associated with KBD. (a) Wilcox-test results for evaluating the relative abundance of significantly different microbiota at the phylum level. (b) Wilcox-test results for evaluating the relative abundance of significantly different microbiota at the genus level.

**Figure S2** Predicted function of gut microbiota based on COGs, EC, KO, PFAM and TIGRFAM analysis. The extended error bar plot showed the top 30 significantly different functions between KBD and NC group. (a) The top 30 COGs between KBD and NC group. (b) The top 30 EC between KBD and NC group. (c) The top 30 KO between KBD and NC group. (d) The top 30 PFAM between KBD and NC group. (e) The top 30 TIGRFAM between KBD and NC group.

**Figure S3** The information of gene catalogue based on the metagenomic sequencing. (a) The distribution of UniqGene length, (b) The dilution curve of core genes, (c) The dilution curve of pan genes. (d-f) Venn diagrams demonstrate the number of altered genes shared among gradeⅠ,ⅡKBD and NC group. (g) he relative abundance of top 20 species enriched in gradeⅠKBD versusⅡKBD. The box represents the interquartile ranges, inner line denotes the median.

**Figure S4** GO function classifcation analysis of differentially expressed unigenes between gradeⅠKBD and NC group (a), gradeⅡKBD and NC group (b) and gradeⅠKBD and gradeⅡKBD group (c). GO enrichment analysis of differentially expressed unigenes between gradeⅠKBD and NC group (d), gradeⅡKBD and NC group (e) and gradeⅠKBD and gradeⅡKBD group (f).

**Figure S5** The aberrant metabolic patterns in KBD (gradeⅠandⅡ ) compared with normal controls. (a) Heat map of the 32 idms2 significantly different metabolites across gradeⅠandⅡ KBD. (b) Heat map of the 10 idms2 significantly different metabolites across gradeⅡKBD and normal controls. Metabolites >2-fold changes, VIP ≥ 1, P < .05 (T test). Correlations between species and metabolites. (c) The top 30 species were detected in Metagenomic data. Metabolites >2-fold changes between gradeⅡKBD and normal controls, with P < .05 (T test), VIP ≥ 1. The correlation effect is indicated by a color gradient from green (negative correlation) to red (positive correlation). * P < .05, ** P < .01, T test. (d) Correlations between species and metabolites. The top 30 species were detected in Metagenomic data. Metabolites >2-fold changes between gradeⅠandⅡKBD, with P < .05 (T test), VIP ≥ 1. The correlation effect is indicated by a color gradient from green (negative correlation) to red (positive correlation). * P < .05, ** P < .01, T test. The correlation network of significantly different metabolites and species (e) across gradeⅡKBD and normal controls, (f) across grade gradeⅠandⅡKBD, abs_rho means abundances correlation coefficient.
