# Supplementary material for: Alterations in the gut microbiota and metabolite profiles of patients with Kashin-Beck disease, an endemic osteoarthritis in China
Source: Cell Death Dis. 2021 Oct 28;12(11):1015. doi: 10.1038/s41419-021-04322-2 (PMC8553765; doi:10.1038/s41419-021-04322-2)
Supplement: Supplementary file 7 — Table S1 [file 41419_2021_4322_MOESM7_ESM.docx]

| Characteristic | KBD | NC | P value |
| --- | --- | --- | --- |
| Subjects (n) | 32 | 35 |  |
| Male/Female | 10/22 | 17/18 | >0.05 |
| Age (mean), years | 62 | 61 | >0.05 |
| Grade Ⅰ | 16* | - | - |
| Grade Ⅱ | 16* | - | - |
| BMI | 23.41 | 23.98 | >0.05 |
| Stiffness | 32 | 4 | <0.05 |
| Arthralgia | 29 | 0 | - |
| Phalanges tuberositas | 16 | 0 | - |
| Brachydactylia | 16 | 0 | - |
| Dyskinesia of wrist joints | 11 | 0 | - |
| Dyskinesia of elbow joints | 7 | 0 | - |
| Dyskinesia of knee joints | 5 | 0 | - |
| Dyskinesia of ankle joints | 9 | 0 | - |
| Fracture | 2 | 0 | - |
| Smoking | 7 | 12 | >0.05 |

Table S1 Characteristics of participants in this study

* grade ⅠandⅡ KBD patient according to the national diagnostic criteria of KBD in China [WS/T 207-2010]
